# 

**Published:** 1891-01

**Authors:** 


					﻿Vol. XL
JANUARY, 1891.
NO. 1.
JRHE
Homeopathic physician,
A Monthly Journal of Homoeopathic Materia Meaica
and Clinical Medicine.
“ .He is a freeman whom truth makes free, and all are slaves beside."
“ Seek the Truth : come whence it may, cost what it will."
EDITED AND PUBLISHED BY
WALTER M. JAMES, Nf. D.,
AND
GEORGE H. CLARK-Afe£C
bLNI's7>
PHILADELPHIA :
No. 11£25 SPKUCK STKEBT.
London
ALFRED HEATH & CO., Sole Agents for Great Britain,
114 Ebury Street, Eaton Square.
Kg-Yearly Subscription, $2.50. invariably in advance. Single number s, kfi rts
Entered at the Philadelphia Peet-Office as 8econ<I-Class Mail Matter.
				

